# Common and diet-specific metabolic pathways underlying residual feed intake in fattening Charolais yearling bulls

**DOI:** 10.1038/s41598-021-03678-x

**Published:** 2021-12-21

**Authors:** Ezequiel Jorge-Smeding, Muriel Bonnet, Gilles Renand, Sébastien Taussat, Benoit Graulet, Isabelle Ortigues-Marty, Gonzalo Cantalapiedra-Hijar

**Affiliations:** 1 Facultad de Agronomía, Departamento de Producción Animal y Pasturas, Universidad de la República, Av. Garzón 780, Montevideo, Uruguay; 2 Université Clermont Auvergne, INRAE, VetAgro Sup, UMR Herbivores, 63122 Saint-Genès-Champanelle, France; 3 INRAE, AgroParisTech, GABI, Université Paris-Saclay, 78350 Jouy-en-Josas, France; 4 Allice, 149 Rue de Bercy, 75595 Paris Cedex 12, France

**Keywords:** Metabolomics, Physiology

## Abstract

Residual feed intake (RFI) is one of the preferred traits for feed efficiency animal breeding. However, RFI measurement is expensive and time-consuming and animal ranking may depend on the nature of the diets. We aimed to explore RFI plasma biomarkers and to unravel the underlying metabolic pathways in yearling bulls fed either a corn-silage diet rich in starch (corn diet) or a grass-silage diet rich in fiber (grass diet). Forty-eight extreme RFI animals (Low-RFI, n = 24, versus High-RFI, n = 24, balanced per diet) were selected from a population of 364 Charolais bulls and their plasma was subjected to a targeted LC-MS metabolomic approach together with classical metabolite and hormonal plasma analyses. Greater lean body mass and nitrogen use efficiency, and lower protein turnover were identified as common mechanisms underlying RFI irrespective of the diet. On the other hand, greater adiposity and plasma concentrations of branched-chain amino acids (BCAA) together with lower insulin sensitivity in High-RFI animals were only observed with corn diet. Conversely, greater plasma concentrations of BCAA and total triglycerides, but similar insulin concentrations were noted in efficient RFI cattle with grass diet. Our data suggest that there are diet-specific mechanisms explaining RFI differences in fattening Charolais yearling bulls.

## Introduction

In a growing world’s human population the efficient use of natural resources becomes a crucial issue for sustainable livestock systems. In growing animals, animal feed efficiency can be defined as the animal ability to reach a market or adult BW with the least amount of feed intake^1^. Animal feed efficiency is quite variable across individuals^2^ and moderately heritable^3^, leading to the opportunity to genetically select animals for this trait with potential impacts on profitability and sustainability of beef production systems^4^. Among the metrics for evaluating animal feed efficiency, residual feed intake (RFI) is one of the more widely used trait within animal breeding, enabling us to identify individuals with lower feed intake but similar performances.

As for any other feed efficiency trait, RFI determination is expensive and time-consuming, needing at least 6 weeks of individual daily feed intake and body weight gain recording^5^. For this reason, research aimed at exploring biological markers of RFI has become of scientific interest in the last years as a way to find easier and cheaper strategies for identifying individuals with superior feed efficiency. Moreover, the discovery of biomarkers may help to gain deeper insights on the physiological mechanisms underlying individual variability in feed efficiency, which may contribute to better define genetic selection programs or precision feeding systems.

Metabolomics methodologies leading to high-throughput phenotyping have been used both to explore biomarkers and to unravel the metabolic mechanisms underlying individual variability of feed efficiency^6,7^. Targeted rather than untargeted metabolomic technologies are usually the best option when the identification of metabolic pathways and the mechanism associated to a particular condition is pursued^8^. However, results have not been consistent across studies, and candidate plasma biomarkers still remain to be validated before being globally accepted and used^9,10^. The lack of universally accepted biomarkers of RFI might be due to the difficulty of identifying metabolites driven by the inter-individual variability rather than by dietary treatments, the latter having a strong impact on plasma metabolome^6,11^. Moreover, the inconsistencies across studies could stem from the use of different types of diets since a potential RFI $\times$ Diet interaction has been evoked in a few works^12,13^. This could mean that biological mechanisms underlying RFI, and thus the associated biomarkers, might differ depending on the nature of the diet and finally to the type of absorbed nutrients. This is a crucial issue if RFI selection programs are to be applied in beef cattle systems where a wide variety of feeding conditions exists.

Therefore, the aim of this work was both exploring plasma biomarkers and unravelling key metabolic pathways that could explain the RFI differences in extreme purebred Charolais yearling bulls, the most representative beef cattle breed in France, assigned to two contrasting fattening diets, either a corn-silage diet rich in starch or a grass-silage diet rich in fiber. To the best of our knowledge, our study presents the largest experimental set up for exploring RFI biomarkers in fattening bulls through a targeted metabolomic approach.

## Results

### Animal performances

As previously reported^14^, the RFI differences between Low and High-RFI animals averaged 1.48 kg DMI. Accordingly, the daily intake of organic matter, crude and metabolizable protein, neutral detergent fiber and net energy were also lower (− 13 % on average, *P* < 0.01) for Low- versus High-RFI animals, irrespective of diet (Table 1). However, starch intake differed between RFI groups in a different way according to the diet (RFI $\times$ Diet; *P* < 0.01) as it was lower for Low- than High-RFI animals only in the corn silage diet (Table 1). Estimated net portal appearance of total volatile fatty acids, acetate, propionate and butyrate were lower (*P* < 0.01) for Low- than High-RFI animals in both diets. In agreement with what was observed for starch intake, the net portal appearance for glucose differed between RFI groups in a diet-dependent manner (RFI $\times$ Diet; *P* < 0.01) as it was lower for Low- than High-RFI animals only in the corn diet (Table 1).

**Table 1:** Nutrients intake and absorption in high versus low residual feed intake (RFI) young bulls fed corn- or grass-silage diets.

	Corn		Grass		SEM	*P*-value		
	High-RFI	Low-RFI	High-RFI	Low-RFI		RFI	Diet	RFI × Diet
**a. Measured nutrient intakes**$^\mathbf {1}$								
OM (kg/day)	9.98	8.55	8.77	7.96	0.330	< 0.01	< 0.01	0.20
CP (kg/day)	1.53	1.30	1.34	1.21	0.051	< 0.01	< 0.01	0.20
NDF (kg/day)	3.49	2.99	4.67	4.24	0.142	< 0.01	< 0.01	0.72
Starch (kg/day)	3.14^a^	2.69^b^	0.39^a^	0.35^a^	0.081	< 0.01	< 0.01	< 0.01
**b. Estimated nutrient intake and absorption**								
NE (Mcal/day)	17.0	14.6	14.9	13.5	0.57	< 0.01	< 0.01	0.20
MP (kg/day)	0.90	0.77	0.77	0.70	0.032	< 0.01	< 0.01	0.20
**Net portal appearance (mmol/h kg BW)**$^\mathbf {2}$								
Total Volatil fatty acids	1371	1175	1376	1247	49.0	< 0.01	0.27	0.34
Acetate	928	795	993	900	34.2	< 0.01	< 0.01	0.42
Propionate	325	278	301	273	11.2	< 0.01	0.08	0.26
Butyrate	73	62	55	50	2.3	< 0.01	< 0.01	0.13
Glucose	− 0.046^b^	− 0.054^a^	− 0.097^a^	− 0.098^a^	0.0005	< 0.01	< 0.01	< 0.01

a,b: Means are compared only within each diet. Different letters means significant differences according to Tukey test (*P* < 0.05).

1: Measures based on DMI and diet composition, *OM* Organic matter intake, *CP* Crude protein, *MP* Metabolizable protein, *NDF* Neutral detergent fibre, *NE* Net energy.

2: Estimated according to Loncke et al.^63^.

Ultrasound subcutaneous fat depth measured at the end of the RFI test on three different anatomical regions was similar (*P* > 0.05) across diets and RFI groups (Table 2). Hot carcass body weight was higher in Low- versus High-RFI animals (+ 6%; *P* = 0.01) for both diets. In contrast, visceral fat mass (kg or % of body weight) was affected by an RFI $\times$ Diet interaction (*P* $\le$ 0.07) with less visceral fat in Low- versus High-RFI animals in the corn diet but with similar levels with the grass diet.

**Table 2:** Animal performances and plasma biochemical parameters in High versus Low residual feed intake (RFI) young bulls fed corn- or grass-silage diets.

	Corn		Grass		SEM	*P*-value		
	High-RFI	Low-RFI	High-RFI	Low-RFI		RFI	Diet	RFI × Diet
**a. Performance and carcass traits**								
Initial BW$^1$ (kg)	390	397	372	367	13.2	0.38	0.26	0.81
Final BW$^1$ (kg)	730	725	650	698	19.1	0.26	0.01	0.19
**Final back fat depth (mm)**								
12–13th rib	2.98	2.68	2.71	2.54	0.331	0.32	0.39	0.77
Lumbar	3.59	3.03	2.93	2.90	0.330	0.21	0.10	0.26
Gluteal	3.36	3.28	3.77	3.19	0.732	0.53	0.75	0.64
Hot carcass weight (kg)	431	444	410	449	10.3	0.01	0.41	0.20
Visceral fat$^2$ (kg)	7.13$^{\rm{a}}$	5.28$^{\rm{b}}$	6.79$^{\rm{a}}$	7.89$^{\rm{a}}$	0.661	0.57	0.09	0.03
Visceral fat$^2$ (%)	0.96$^{\rm{a}}$	0.73$^{\rm{b}}$	1.00$^{\rm{a}}$	1.10$^{\rm{a}}$	0.083	0.34	0.01	0.07
**b. Metabolites and hormones**								
Urea (g/L)	0.21	0.18	0.15	0.12	0.010	0.01	< 0.01	0.82
NEFA (mol/L)	0.121	0.121	0.122	0.153	0.0182	0.40	0.36	0.37
BHB (mol/L)	0.344	0.311	0.238	0.241	0.0201	0.45	< 0.01	0.36
Glucose (g/L)	0.77	0.79	0.66	0.71	0.031	0.29	< 0.01	0.51
Insulin ($\upmu$IU/ml)	30.7$^{\rm{a}}$	20.7$^{\rm{b}}$	13.4$^{\rm{a}}$	15.7$^{\rm{a}}$	3.03	0.19	< 0.01	0.04
IGF-1 (ng/mL)	456	430	398	444	25.4	0.70	0.38	0.16
RQUICKI	0.28$^{\rm{b}}$	0.33$^{\rm{a}}$	0.36$^{\rm{a}}$	0.31$^{\rm{a}}$	0.021	0.79	0.16	0.02
**c. Vitamins**								
FAD ($\upmu$mol/L)	0.22	0.22	0.22	0.22	0.009	0.64	0.99	0.58
Ribo ($\upmu$mol/L)	0.02	0.02	0.02	0.02	0.002	0.84	0.40	0.51
B2 total ($\upmu$mol/L)	0.24	0.23	0.24	0.24	0.010	0.70	0.87	0.69
FAD:B2 total	0.94	0.93	0.93	0.93	0.042	0.73	0.31	0.37
Ribo:B2 total	0.06	0.07	0.07	0.07	0.013	0.73	0.31	0.37
FAD:Ribo	15.0	14.0	14.0	14.2	1.21	0.68	0.60	0.50
P5P ($\upmu$mol/L)	0.38	0.39	0.38	0.33	0.030	0.49	0.13	0.14
PA ($\upmu$mol/L)	0.028	0.029	0.025	0.027	0.0021	0.29	0.02	0.70
B6 total ($\upmu$mol/L)	0.41	0.42	0.40	0.36	0.030	0.55	0.11	0.16
P5P:B6 total	0.93	0.93	0.94	0.93	0.004	0.28	0.79	0.13
PA:B6 total	0.07	0.07	0.06	0.07	0.004	0.28	0.79	0.13
P5P:PA	13.7	14.4	15.8	13.2	0.12	0.33	0.63	0.09

a,b: Means are compared only within each diet. Different letters means significant differences according to Tukey test (*P* < 0.05).

1: Body weight at the begining and the end of the RFI test.

2: Kindey, pelvic and mesenteric fat.

3: *NEFA* Non-esterified fatty acid, *BHBA* $\beta$-hydroxybutyrate, *IGF-1* Insulin growth factor-1; RQUICKI index.

### Plasma concentrations of metabolites, hormones and vitamins

Among the classical plasma biochemical parameters here analyzed, only plasma urea concentration differed across RFI groups, being greater for High- versus Low-RFI animals (+ 21%; *P* = 0.01; Table 2). In relation to the diet effect, the corn diet promoted greater (*P* < 0.01) plasma concentrations of glucose, urea and $\beta$-hydroxbutyrate (BHB) compared to the grass diet. Differences across RFI groups on plasma insulin levels and the Revised Quantitative Insulin Sensitivity Check Index (RQUICKI) were diet-dependent (RFI $\times$ Diet; *P* $\le$ 0.04) with lower values in efficient versus inefficient RFI animals only when they were fed the corn diet. As expected, higher insulin levels were found in corn versus grass-based diets (*P* < 0.01). No effect of diet or RFI group on plasma IGF-1 concentration was found (*P* > 0.05). Blood B vitamins concentrations here analyzed, vitamins B2 and B6 sums as well as ratios between vitamins, were not statistically different across RFI groups (*P* > 0.05; Table 2). However, the plasma concentrations of pyridoxal (PA) were significantly lower for animals fed grass than for those fed corn (− 11%; *P* < 0.02).

### Metabolomic profiling and multivariate analysis

**Figure 1:**
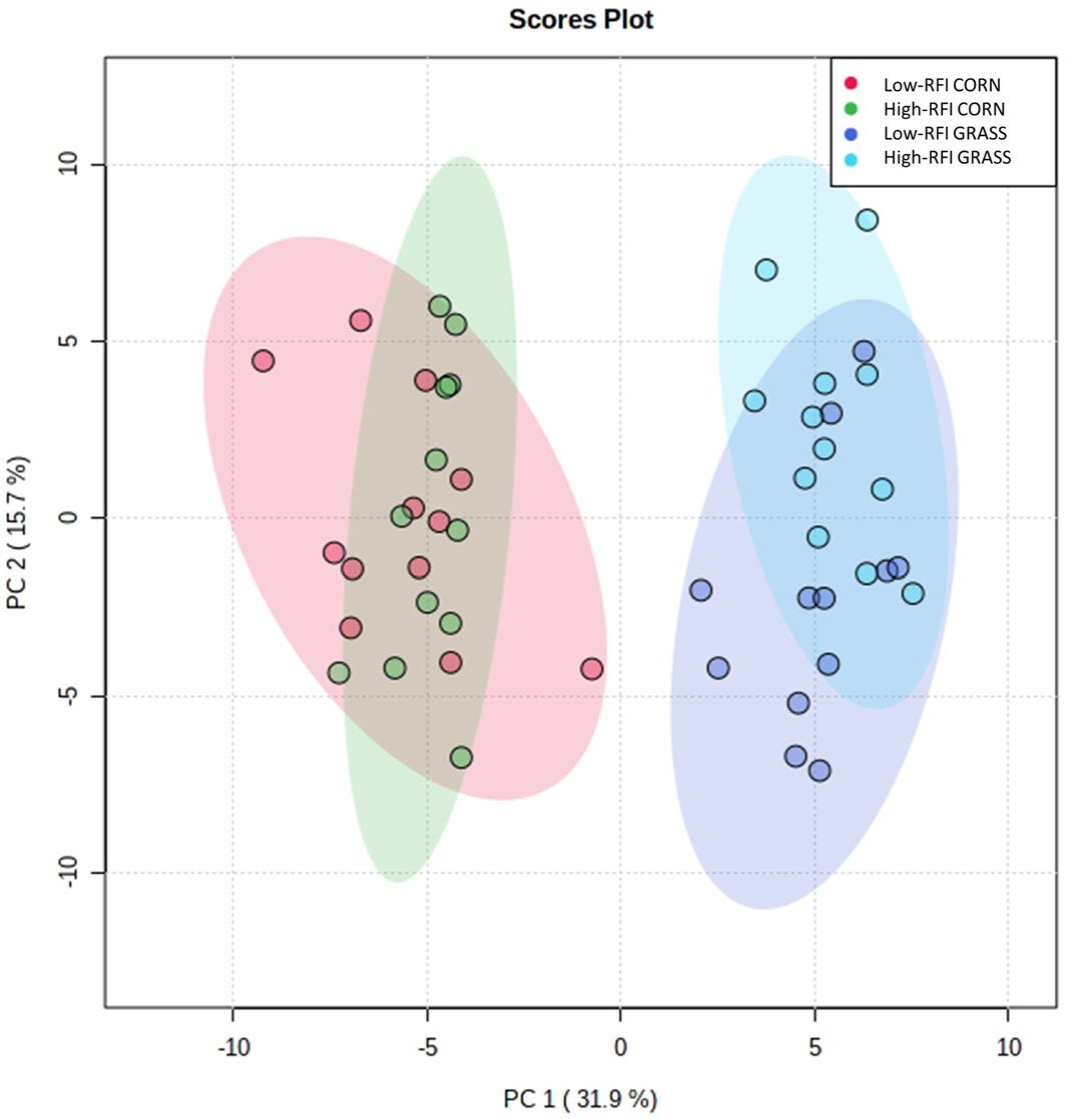
Score plots for principal components analysis (PCA) comparing Low-RFI versus High-RFI animals fed corn- or grass-silage diets based on all quantified metabolites

Only 271 out of 630 metabolites assayed (Supplementary Table S1 online) by the MxP$^\circledR$ Quant 500 kit were effectively quantified (i.e. metabolite plasma concentration greater than the limit of detection in at least 80 % of the 48 plasma samples, Supplementary Table S2 online). The principal component analysis (PCA) performed on these 271 metabolites clearly discriminated individuals according to the diet they were fed on, but failed to discriminate individuals according to their RFI group (Fig. 1). According to partial least squares discriminant analysis (PLS-DA), no model for predicting the RFI group was obtained for the corn diet (*Q*$^2$ = − 0.07, 1st component model; Supplementary Fig. S1 online) and only a poor predictive model was obtained for grass diets (*Q*$^2$ = 0.30, *R*$^2$ = 0.54 for 1st component model; Supplementary Fig. S1 online). Consequently, no RFI discriminant metabolites based on variable importance in projection (VIP) values could be proposed at this step.

Detailed information on the 271 metabolites identification, their plasma concentrations and ANOVA statistics on experimental factors is available in the Supl. Table S2. ANOVA revealed that creatinine, $\beta$-alanine, trans-4-hydroxyproline, p-cresol sulphate and three specific triglycerides (TG) (TG17:0_34:1, TG16:0_33:1 and TG20:3_34:0) were increased while phosphatidylcholine (PC) *ae* 30:1 was decreased (*FDR* < 0.05) in Low- versus High-RFI bulls irrespectively of the diet (Table 3).

**Table 3:** Blood plasma concentration of metabolites quantified through LC-MS and differing (FDR < 0.05) between extreme RFI young bulls (Low versus High RFI) fed either corn- or grass-silage diets.

	Corn$^1$		Grass$^1$		SEM	RFI	Diet	RFI $\times$ Diet
	High-RFI	Low-RFI	High-RFI	Low-RFI				
Leu$^2$	133$^{\rm{a}}$	106$^{\rm{b}}$	104$^{\rm{a}}$	116$^{\rm{a}}$	15.4	0.36	0.07	0.01
Val	234 $^{\rm{a}}$	205$^{\rm{b}}$	192$^{\rm{b}}$	217$^{\rm{a}}$	21.6	0.90	0.04	0.01
Ile	128$^{\rm{a}}$	110$^{\rm{b}}$	110$^{\rm{a}}$	124$^{\rm{a}}$	14.1	0.75	0.65	0.04
PC ae C38:2	2.83$^{\rm{a}}$	2.49$^{\rm{a}}$	3.18$^{\rm{b}}$	3.89$^{\rm{a}}$	0.441	0.72	< 0.01	0.03
Creatinine	183	218	152	185	22.9	< 0.01	< 0.01	0.79
Trans-4-Hydroxyproline	37.1	44.0	38.6	42.7	5.42	0.05	1.00	0.53
$\beta$-Alanine	2.03	2.60	1.91	2.38	0.501	0.05	0.32	0.80
p-Cresolsulphate	60.4	68.1	48.2	56.2	8.23	0.05	< 0.01	0.78
PC ae C30:1	0.83	0.60	1.27	1.01	0.256	0.04	< 0.01	0.54
TG17:0_34:1	0.55	0.51	0.37	0.53	0.130	0.04	0.16	0.14
TG16:0_33:1	0.50	0.49	0.43	0.60	0.118	0.05	0.92	0.13
TG20:3_34:0	0.24	0.26	0.23	0.25	0.110	0.05	< 0.01	0.78

a,b: Means are compared only within each diet. Different letters means significant differences according to Tukey test (*P* < 0.05).

1: Plasma concentration (μM) are presented as mean and SEM.

2: Metabolite’s names for these acronyms are available in Supplementary Table S1.

In contrast, the three BCAA (isoleucine, leucine, and valine) as well as PCA ae 38:2 were significantly affected by RFI (Table 3) but in a different way according to the type of diet (RFI × Diet; *FDR* < 0.05). The plasma concentration of the 3 individual BCAA was greater for High- versus Low-RFI in the corn diet but similar in the grass diet, except for valine showing significant greater concentration in Low- versus High-RFI in the grass diet. Conversely, the PC ae 38:2 was greater for Low- versus High-RFI animals only in the grass diet.

In order to gain a better understanding, the sums of key metabolic families as well as some ratios between metabolites were calculated *a posteriori* (Supplementary Table S2). Among them, only the sum of non-essential amino acids (NEAA) was significantly affected by RFI (*FDR* < 0.05) as it was greater for Low- versus High-RFI animals irrespective of the diet. In addition, only total BCAA were affected by the RFI × Diet interaction (*FDR* = 0.01), as total BCAA were greater for High- versus Low-RFI animals in corn diet, but lower in grass diet. Total triglycerides also tended to be affected by the RFI × Diet interaction (*FDR* = 0.06) as they were greater for Low- versus High-RFI animals only in the grass diet.

**Figure 2:**
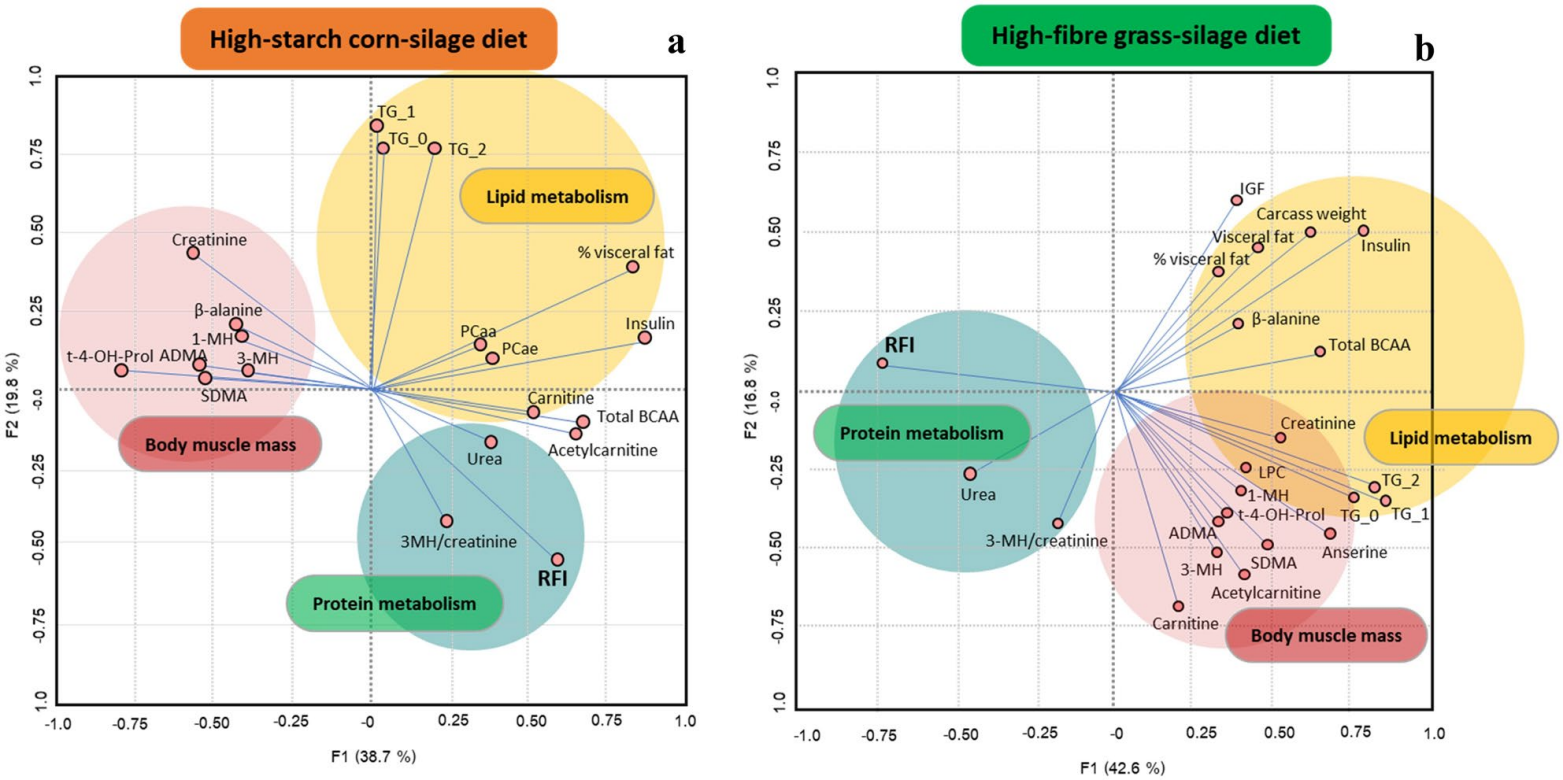
Loading plots of principal component analysis conducted a posteriori according to variables highly involved in shared and diet-dependent metabolic pathways underlying RFI variation in corn- (**a**) and grass-silage (**b**) diets. TG_n denote triglycerides with increasing saturations (0, 1, or more for TG_0, TG_1 and TG_2, respectively); t-4-OH-Prol: trans-4-hydroxyproline; 3MH/creatinine: 3-methylhistidine to creatinine ratio; 1-MH: 1-methylhistidine, 3-MH: 3-,methylhistidine; SDMA: symmetric dimethyl arginine; ADMA: asymmetric dimethyl arginine; PCaa: total diacyl-phosphatidylcholine, PCae: total acyl-ethyl-phosphatidylcholines

Results from a PCA performed *a posteriori* only from those variables significantly different across RFI (Fig. 4) indicated that plasma concentration of insulin and BCAA, and visceral fat mass were positively correlated with RFI in the corn diet (PCA first component; 38.7%). In contrast, these same variable as well as plasma triglycerides showed a negative association with RFI in the grass diet (PCA first component; 42.6%) (Fig. 2).

### Metabolic pathway analysis

**Figure 3:**
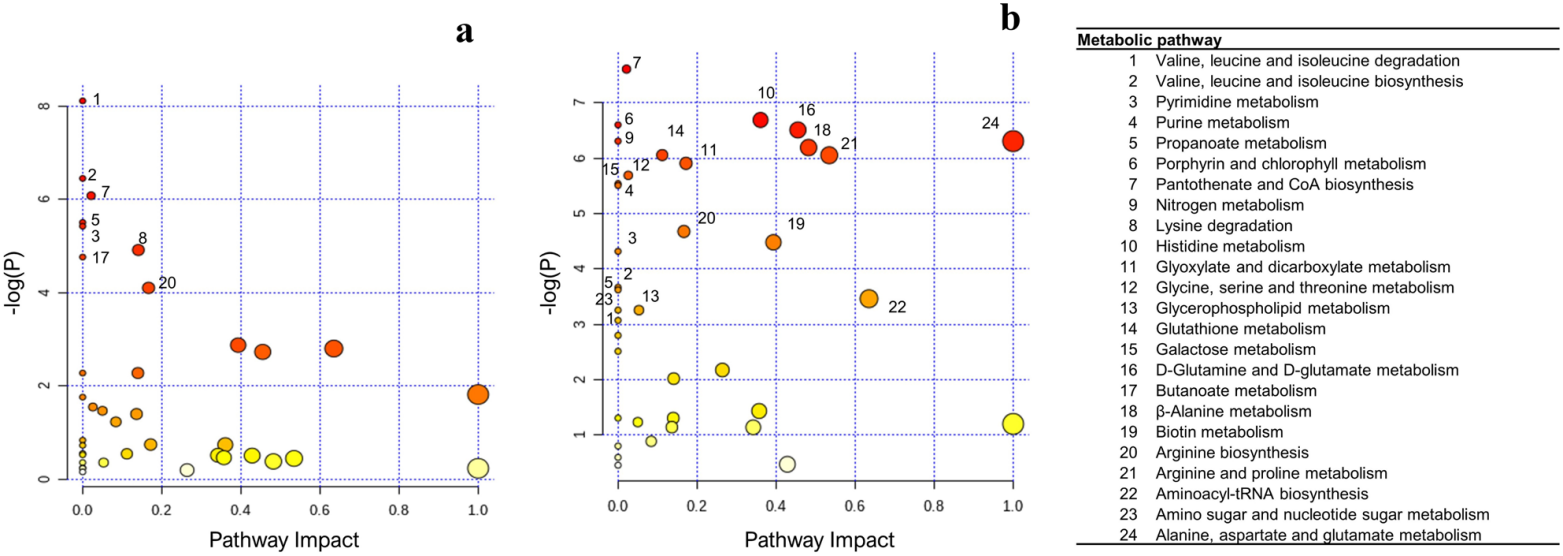
Metabolic pathway analysis comparing Low versus High-RFI animals in corn- (**a**) and grass-silage (**b**) diets according to *Bos taurus* KEGG database. Numbers indicate metabolic pathways differing between RFI groups within each given diet. More red tones denote lower *P* values, while greater circle sizes denote greater impact values

**Figure 4:**
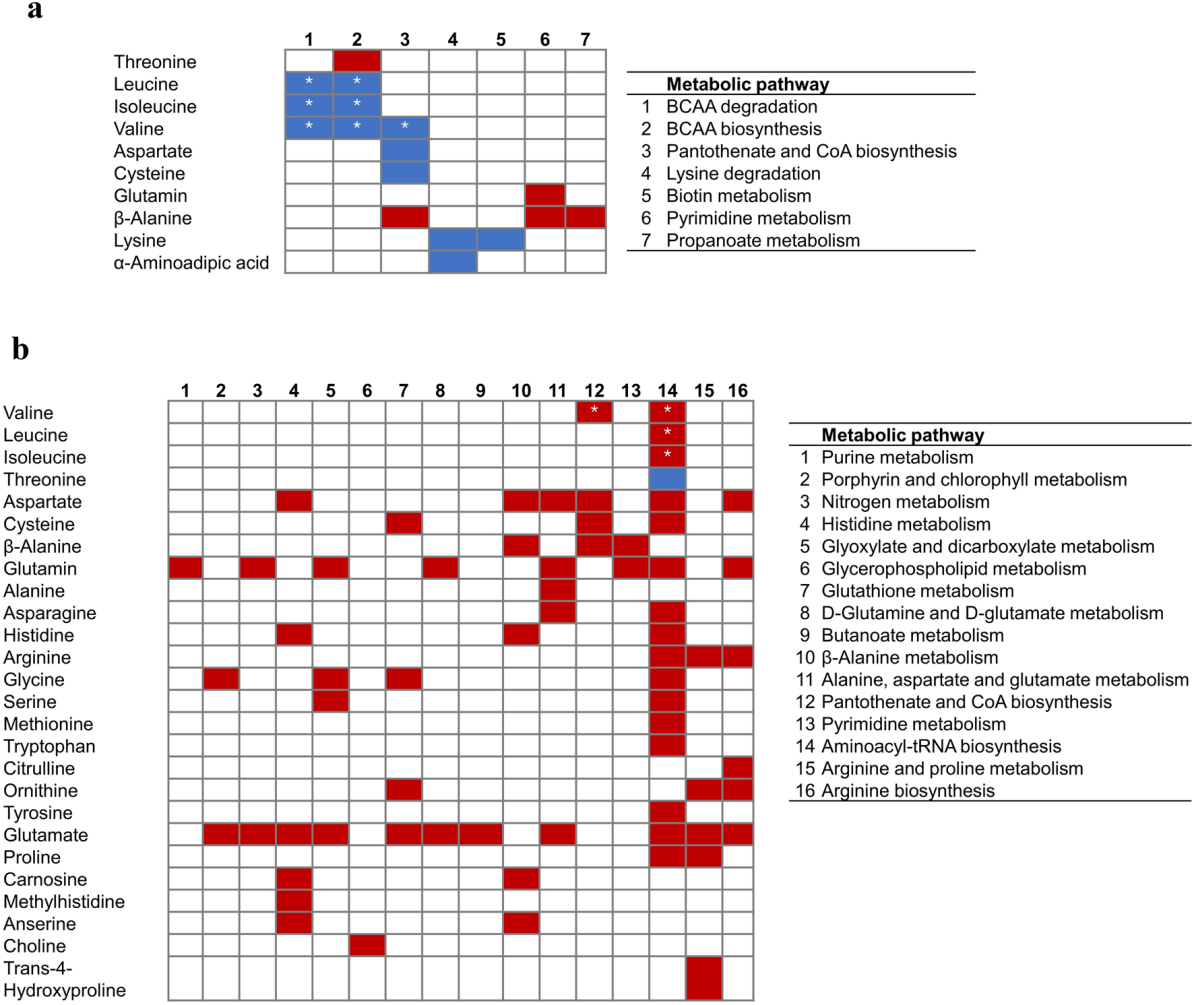
Effectively detected and quantified metabolites involved in metabolic pathways differing between RFI groups fed the corn (**a**) or the grass (**b**) diet. Metabolic pathways references are stated next to the correspondent panel. Significant differences (Tukey’s test < 0.05) between Low versus High-RFI within the given diet are depicted with *, whenever RFI × Diet interaction significant effect (*FDR* < 0.05 in the ANOVA analysis). Cells’ color code depict metabolites concentrations for Low- versus High-RFI animals (red: increased, blue: decreased)

Metabolic pathway analysis revealed 7 and 16 metabolic pathways differing (*FDR* < 0.05) between RFI groups in the corn and grass diets, respectively (Table 4, Fig. 3). Among these metabolic pathways, pantothenate and CoA biosynthesis, and pyrimidine metabolism were shift-regulated between RFI groups in both diets. While pyrimidine metabolism was up-regulated in Low-RFI animals in both diets, pantothenate and CoA biosynthesis was down-regulated in the corn diet and up-regulated in the grass diet (Fig. 4). In the corn diet, most of metabolic pathways were associated with lower plasma concentration of metabolites in Low-RFI such as those involved in BCAA metabolism, pantothenate and CoA biosynthesis, lysine degradation and biotin metabolism (Fig. 4). The opposite trend was found for the grass diet, where most of the metabolites involved in the identified metabolic pathways had greater plasma concentrations for Low-RFI than High-RFI animals (Fig. 4).

**Table 4:** Identified metabolic pathways associated with RFI differences in young bulls fed either corn- or grass-silage diets.

Pathway	TC^1^	Hits^2^	Corn		Grass^1^	
			FDR^3^	Impact^4^	FDR	Impact
$\beta$-Alanine metabolism	21	5	–	–	0.01	0.5
Valine, leucine and isoleucine degradation	40	3	0.01	0.00	–	–
Valine, leucine and isoleucine biosynthesis	8	4	0.03	0.00	–	–
Pyrimidine metabolism	38	2	0.03	0.00	0.03	0.00
Purine metabolism	66	1	–	–	0.01	0.00
Propanoate metabolism	23	1	0.03	0.00	–	–
Porphyrin and chlorophyll metabolism	30	2	–	–	0.01	0.00
Pantothenate and CoA biosynthesis	19	4	0.03	0.02	0.01	0.02
Nitrogen metabolism	6	2	–	–	0.01	0.00
Lysine degradation	25	2	0.05	0.14	–	–
Histidine metabolism	16	6	–	–	0.01	0.36
Glyoxylate and dicarboxylate metabolism	32	5	–	–	0.01	0.17
Glycerophospholipid metabolism	36	1	–	–	0.01	0.03
Glutathione metabolism	28	4	–	–	0.01	0.11
D-Glutamine and D-glutamate metabolism	5	2	–	–	0.01	1.00
Butanoate metabolism	15	1	–	–	0.01	0.00
Biotin metabolism	10	1	0.05	0.00	–	–
Arginine biosynthesis	14	6	–	–	0.01	0.48
Arginine and proline metabolism	38	5	–	–	0.03	0.39
Aminoacyl-tRNA biosynthesis	48	20	–	–	0.03	0.17
Alanine, aspartate and glutamate metabolism	28	5	–	–	0.01	0.53

1: Total number of metabolites theoretically considered by the KEGG database for identify metabolic pathways.

2: Metabolites effectively quantified in the current study (Figs. 3, 4).

3: FDR > 0.05 is depicted by “–”.

4: Topological analysis of impact of the current metabolic pathway.

## Discussion

Before implementing genetic programs for improving animal feed efficiency several issues should be first addressed. One is related to the potential interaction between genetics and environment (G$\times$ E), such that animals with superior performance may not be the same across different environments. For instance, this may be a concern for improving feed efficiency through selection if the animal ranking changes with the quality of the offered diet, the latter differing highly across beef production systems. In this sense, several studies have pointed at a re-ranking of beef cattle in terms of RFI when energy-dense diets were shifted to more grass-based diets or vice versa^13,15^. In this context, the aim of our study was to use targeted metabolite analysis to evaluate the biological mechanisms underlying RFI variation in beef cattle fed a classical energy-dense fattening diet based on corn silage (high-starch) versus a grass silage diet (high-fiber) . Our results suggest that both shared and diet-specific mechanisms underlying RFI may exist. As further discussed, some interactions between RFI and the type of diet for several plasma metabolites involved in protein and lipid metabolic pathways were observed. On the other hand, many common metabolites and pathways were found to diverge between RFI groups across the two diets suggesting that some features and process such as body composition, muscle protein degradation and N use efficiency are likely common determinants of RFI variations irrespective of the diet.

Among the few metabolites showing FDR significant differences across RFI groups, and irrespective of the diet, there were creatinine and trans-4-hydroxyproline (concentrations + 16 % for Low- versus High-RFI on average). These two metabolites reflect directly or indirectly changes in muscle body mass. Creatinine, is a breakdown product of creatinine phosphate in muscle^16^, and its plasma/blood concentration has been proposed and used as a proxy of muscle mass in ruminants^17^. The higher plasma values of creatinine in Low-RFI animals for both diets agree with previous reports in beef cattle^9,15,18^ indicating higher muscle body mass in efficient versus inefficient cattle. Likewise, trans-4-hydroxyproline, a major component of collagen, showed higher plasma concentration in efficient RFI animals. Plasma trans-4-hydroxyproline has been proposed as a biomarker of total collagen body mass^19^, and thus its plasma concentration might indirectly reflect differences in the amount of connective tissues associated to the musculoskeletal system. In this regard, plasma trans-4-hydroxyproline was found in greater concentration in beef steers with higher growth rate and lean mass^20^. Additionally, $\beta$-alanine was also consistently higher in Low-RFI animals across the two diets. Even if it is considered to be at the crossroad of many metabolic pathways, higher $\beta$-alanine (i.e. a breakdown product from the muscle-rich dipeptides anserine and carnosine) in plasma of Low-RFI animals might also agree with greater body muscle mass. According to a recent review, RFI trait is negatively associated with lean body mass^1^ and recent genetic studies demonstrated that carcass muscle content was negatively correlated to RFI in Charolais bulls^21^. Taken together, the differences in plasma concentration across RFI groups for these three endogenous metabolites support a contrasted body composition across RFI groups whatever the diet is used. However, it is noteworthy to mention that these differences in body composition were not reflected by the real-time ultrasound echography in our conditions (i.e. late maturing breed). Our findings highlight the need for other proxies of body composition in Charolais yearling bulls to be used in the RFI model.

Because efficient RFI animals eat less protein (as here reported) but retain equal (or greater) amount of protein^22^ it could be expected that the N use efficiency (i.e. N retention/N intake) will be improved. This has been empirically confirmed in dairy cows with contrasted RFI values^23^. However, milk N secretion is easier to measure than N accretion in growing animals. In growing cattle, most studies have failed to prove this link through N balance measurements^24,25^. However, results from our team using a novel isotopic approach applied to this same experimental setup (n = 48) suggested that more efficient animals in terms of RFI also show higher N use efficiency compared to less efficient RFI animals^26^. Data from the present study showed that plasma urea concentration, a confirmed biomarker of N use efficiency^27^, was decreased in more efficient animals in agreement with some reports^18,22,28^ but unlike others^6,29^. Interestingly, a non-targeted metabolomics approach applied to the plasma of growing beef heifers revealed the urea cycle as a metabolic pathway underlying RFI differences^30^. Furthermore, the metabolic pathways analysis identified the pyrimidine metabolism as the unique pathway shifting across RFI groups in the same way in both diets. Pyrimidine biosynthesis is intimately linked to the urea cycle pathway through carbamoyl phosphate and pyrimidine catabolism and it has been suggested to contribute to the N use efficiency in dairy cows^31^.

Despite our observation that efficient RFI animals had a greater muscle mass, our results indicated a trend for a lower fractional protein degradation rate of skeletal muscle as indicated by the lower plasma 3-methylhistidine to creatinine ratio (*FDR* = 0.10; Supplementary Table S2). Plasma 3-methylhistidine to creatinine ratio has been reported to be a useful index to evaluate myofibrillar protein degradation^32^ and creatinine allows normalizing by unit of muscle mass. Similar studies looking at this ratio in urine from beef cattle did not, however, find any difference and concluded that the muscle degradation rate was similar across RFI groups^18,33^. In contrast, evidence of lower protein turnover rate in efficient RFI animals have been reported in cattle^34,35^ and swine^36^ using indirect molecular approaches. Similarly, Carvalho et al.^37^ found greater muscle abundance of Heat Shock Protein $\beta$1 (HSPB1) in skeletal muscle for Low- than High-RFI animals and suggested this difference may account for an increased actin and myosin degradation and protein breakdown, and therefore greater protein turnover in skeletal muscle of less efficient animals. Because of the energy cost of protein turnover, our results would agree with the fact that Low-RFI animals have a lower metabolic rate and energy expenditure compared to High-RFI animals^1^.

Finally, the p-cresol concentrations were greater in Low- versus High-RFI animals regardless the diet. P-cresol is a rumen metabolite of tyrosine^38^ which may further undergo a sulphation reaction by the host’s cells into p-cresol sulphate^39^. Higher plasma levels of p-cresol sulphate in efficient RFI animals may translate a higher rumen fermentation rate of feed proteins (tyrosine) because of their expected higher rumen retention time^1^.

Besides common candidate biomarkers of RFI regardless of the diet, our data strongly suggest that there may exist diet-specific metabolic pathways underlying RFI. Interestingly, among the only 4 metabolites showing significant RFI $\times$ Diet interactions, three of them belonged to the family of BCAA (Ile, Leu and Val; *FDR* < 0.05). In this regard, a meta-analysis on genomic regions associated to RFI in beef cattle, mainly fed high-energy dense diets, concluded that the only and unique significant pathway underlying RFI differences was related to BCAA degradation^40^. Similarly, Foroutan et al.^10^ recently reported greater plasma concentrations of BCAA for High- than Low-RFI Angus steers when they were fed on an energy-dense diet. Despite that the first step in BCAA degradation relies on transaminases using B6 vitamin as cofactor^41^ no changes in blood concentration of B vitamin across RFI groups were found in the present study. However, other relevant data potentially related to BCAA metabolism showed a similar RFI $\times$ Diet interaction (i.e. insulin, fat visceral mass) than those observed for BCAA. Results from a PCA only including the most significant biological variables explaining RFI differences (Fig. 4) showed that whereas visceral fat mass and plasma concentration of BCAA and insulin were positively correlated with RFI in the corn diet, all of them along with plasma triglycerides were negatively correlated with RFI in the grass diet. Therefore, cause and effect relationships between RFI, BCAA metabolism, and energy and lipid metabolism, remain unclear.

Our data strongly suggest there may exist diet-specific metabolic pathways underlying RFI. While greater amino acid absorption was estimated in less efficient RFI animals irrespective of the diet (i.e. higher theoretical MP intake), only greater net portal absorption of glucose was estimated in efficient RFI animals when fed the corn diet (Table 1). It seems, thus, that differences in insulin sensitivity associated with RFI would only appear when providing glucogenic and high starch diets. Indeed, the lowest RQUICKI score values observed for High-RFI animals fed the corn diet are indicative of lower insulin sensitivity in these animals^42^. This agrees with previous reports^43,44^ showing that inefficient animals may require more insulin for the uptake of glucose by peripheral tissues compared to the efficient ones. Insulin together with BCAA concentrations, two cell-exogenous signals translating greater energy and AA availability, are known to jointly upregulate the mechanistic target of rapamycin (mTOR), which is a master regulator of cell growth and metabolism involved in protein and lipid synthesis^45^. It is important to mention that the mTOR upregulation depends on the synergy of both nutrient signals (insulin and BCAA)^46^ and thus could explain the observed contrasted BCAA profile across RFI phenotypes depending on the nature of diet (glucogenic vs. ketogenic). Although the exact mechanisms linking BCAA plasma concentrations and insulin sensitivity are far from being well understood in ruminants, it seems reasonable to infer from human studies^47^ that this link is regulated through the mTOR pathway. In this sense, the mTOR pathway has been previously demonstrated to be upregulated in tissues from inefficient RFI beef cattle fed corn-based diets^35^. Our data can be interpreted as the greater glucogenic nutrients and BCAA absorption in High-RFI animals fed the corn diet would have led to a chronic activation of the mTOR pathway and thus to a lower insulin sensitivity^48,49^ and higher BCAA catabolism. Catabolic intermediates of BCAA could have promoted lipogenesis in High-RFI animals fed the corn diet^50^, and thus contributing with insulin to explain their higher visceral fat mass, when compared to Low-RFI animals. Taken together, our results lead us to believe a key role of BCAA and insulin sensitivity for explaining the diet-specific effects on the link between body composition and RFI phenotype.

Unlike what was observed in the corn-based diet, three specific features were noted for the grass-based diet when comparing extreme RFI groups: similar plasma insulin concentration (and likely insulin sensitivity as regarded by the RQUICKI index) and visceral fat mass across RFI groups, but greater plasma concentration of total triglycerides in more efficient animals. Few studies evaluated body composition in contrasted RFI animals fed high-fiber diets. However, among them none have reported significant differences in fat thickness and body condition score across RFI groups^15,51^ unlike what it is usually observed with more energy-dense diets^52,53^. Previous results from our team also pointed at similar visceral fat mass and carcass fat score across divergent RFI animals fed high-fibre diets^6^ which may support the concept of contrasted adiposity across RFI groups depending on the diet. Interestingly, Trujillo et al.^54^ observed that Low-RFI compared to High-RFI heifers had a greater proportion of body fat when animals were tested in grazing conditions. In an experiment with divergent RFI lines in pigs fed two contrasted diets (low vs. high fibre) a significant trend was observed for perirenal fat content to be higher in low versus high-RFI pigs when using high-fibre, high-fat diets whereas the opposite was observed with low-fibre, low-fat diets^55^.

Lastly, regarding the use of plasma metabolites to assist genetic selection, it is important to mention that those metabolites which were found to be affected by the RFI group in a diet-dependent manner should not be considered as universal biomarkers in genetic selection programs. However, several metabolites (e.g.: creatinine. trans-4-hydroxyproline, $\beta$-alanine, p-cresolsulphate) were found to be different across RFI groups regardless the diet. Therefore, we think these metabolites may be potential candidates for biomarker-assisted genetic selection strategies. Further studies across different breeds and diets are required in order to validate these candidate RFI biomarkers. It is noteworthy to say that, alongside with metabolomics, arising technologies are showing that miRNA and mRNA can be useful for feed efficiency biomarkers discovery in cattle^56^. In this sense, it could be imagined that future studies will combine metabolomics and genomic analysis, as complementary approaches rather than exclusive technologies to search biomarkers.

## Conclusions

Although common metabolic pathways underlying RFI were found irrespective of the diet consumed, our data also suggests that there are diet-specific mechanisms explaining RFI differences in Charolais yearling bulls. Among shared mechanisms, we identified muscle mass, protein metabolism and the N use efficiency as the most important common drivers of RFI for both high-starch and high-fibre diets. Concerning diet-specific mechanisms underlying RFI we identified that the pathway linking BCAA catabolism and lipid metabolism was associated to RFI in a diet-dependent manner: when using the high-starch diet the contrasted insulin plasma concentration and sensitivity across RFI groups seemed be positively associated with BCAA plasma concentration and adiposity, while the opposite was observed in the grass diet. We speculate that the mTOR pathway could explain many of the RFI $\times$ Diet interactions here observed. More studies are warranted to confirm our results, especially in other breeds and production systems, and investigate the role of BCAA catabolism and mTOR pathways in the contrasted body composition observed across RFI groups when nutrient supply varies.

## Methods

This study was carried out in compliance with the ARRIVE guidelines and the French legislation on animal care. All procedures were carried out in accordance with relevant guidelines and regulations, approved by the regional ethics committee (Auvergne-Rhône-Alpes, France) and subsequently validated by the French Ministry of Agriculture under the authorization number APAFIS$\#$2930-2015111814299194v3.

### Feed efficiency test and experimental diets

This study is part of a large program aiming to explore plasma biomarkers of RFI in Charolais beef cattle. Details on the feed efficiency test, experimental diets and basic data on animal performances as DM intake, average daily gain and feed efficiency ranking have been previously reported^26^. Briefly, this study used 364 pure bred Charolais young bulls (380 ± 58 kg body weight) tested in 3 different experimental farms. The feed efficiency trials lasted on average 210 ± 16 d (mean ± SD) and were conducted between 2015 and 2018 in 7 independent cohorts (farms $\times$ period) hosting between 48 and 63 young bulls each one. Animals were housed indoors in pens of 5 to 8 animals of similar body weight and evenly allocated to either a high-starch corn silage diet (corn diet) or a high-fibre grass silage diet (grass diet) as previously detailed^26^. Diets were similar between farms and were formulated with a forage to concentrate ratio close to 65:35, providing a minimum of around 1.50 Mcal NE /kg DM, and meeting the recommended metabolizable protein to net energy ratio according to the INRA feeding system^57^. Feed were offered in two daily meals as total-mixed rations. The individual dry matter intake (DMI) was recorded daily using automatic recording troughs (Biocontrol, Rakkestad, Norway). The body weight was determined fortnightly at 0200 pm. The average daily gain (ADG) of each animal was calculated as the slope of body weight regressed on time. Ultrasound echography (Easi-Scan, BCF Technology Ltd., Vienne, France; equipped with a linear probe) was conducted in three different anatomical regions at end of the RFI test as previously described^14^. Distances analyzed from each anatomical place were the skin (D0) and back fat thickness (D1) that include both subcutaneous tissues and skin. The thickness of subcutaneous adipose tissue and connective tissues was calculated as D1-D0. The RFI was calculated as the difference between the actual and predicted DMI. Predicted DMI was calculated based on the ADG, the mean metabolic body weight and the effect of the contemporary group^58^, the latter defined at the level of the pen within each cohort. No significant effects were found for the back fat depth to predict DMI variation beyond the effects of mean metabolic body weight and ADG and thus this variable was not included in the final RFI model.

### Blood sampling

Because the ultimate objective of our research program was to explore RFI biomarkers to assist genetic selection programs, blood was not sampled at the end of the RFI test to be able in field conditions to identify and select future sires long before slaughters. Therefore, blood samples were obtained from each animal one month before the end of each RFI test, which corresponds to an average age of 17.2 months (± 0.51), blood samples were obtained from each animal to explore RFI biomarkers. Blood samples (9 mL) were taken by coccygeal venipuncture using heparinized tubes (BD Vacutainer, Plymouth, UK) before the meal distribution in the morning. Samples were immediately centrifuged (2500 $\times$ g, 15 min, $4\,^{\circ }$C), and the plasma was harvested and stored at $-\,80\,^{\circ }$C until analysis.

To increase the chance of finding RFI biomarkers, analyses were conducted only from 48 extreme animals in terms of RFI values (12 Low-RFI and 12 High-RFI per diet) as usually performed in studies aiming at exploring biomarkers^9,59^. Moreover, to minimize the strong cohort effect on plasma metabolome^6^, the 48 extreme RFI animals were selected from 3 out 7 cohorts while maximizing the RFI differences across RFI groups. Our selection was balanced for the effects of diet, cohort and RFI group and so included 4 animals per diet $\times$ cohort $\times$ RFI group condition. As previously reported^26^, the RFI differences between Low and High-RFI animals averaged 1.48 kg DMI (− 0.71 vs. 0.78 kg DMI of RFI in corn diet for Low and High-RFI respectively, − 0.78 versus 0.77 kg DMI of RFI in grass diet for Low and High-RFI , respectively).

### Animal performances at the slaughterhouse

Animals from the same cohort were slaughtered in the same commercial slaughterhouse when the pen they belonged to approached an average body weight of 720 kg and corresponding to a target market carcass weight of around 430 kg. This corresponded to 22 animals slaughtered right at the end of the RFI test, mostly those fed the corn diet, and the remaining 26 slaughtered several days thereafter (50 ± 14 d) when they reached the targeted body weight. The number of animals within each cohort slaughtered at each time was completely balanced for the RFI groups. Thus, performances at the slaughterhouse were compared at similar body weight and no corrections were performed for differences in age or fattening length. Weights of hot carcass and visceral fat mass (kidney, pelvic and mesenteric fat together) were recorded for each animal.

### Plasma concentrations of metabolites, hormones and vitamins

Blood plasma samples were subjected to spectrophotometrical quantification of glucose (glucose oxidase method), urea (glutamate dehydrogenase method), non-esterified fatty acids (NEFA, acyl-CoA synthase method) and BHB (D-$\beta$-hydroxbutyrate-dehydrogenase method). Determinations were carried out with commercial kits (Thermo Scientific References $\#$981379, $\#$984325 and $\#$981818 for glucose, BHB and urea, respectively and Sobioda Reference $\#$W1W434-91795 for NEFA) using an autosampler spectrophotometer (Arena 20XT, Thermo Fisher Scientific, Cergy Pontoise, France). Intra- and inter-assay coefficients of variation were 1.4 and 3.1% for glucose, 5.9 and 8.5% for urea, 2.1 and 3.0% for NEFA, 4.5 and 5.5% for BHB. Plasma insulin (mean intra-assay coefficients of variation were 6.9% for 5.88 $\upmu$UI/mL and 1.8% for 36.7 $\upmu$UI/mL; Porcine Insulin RIA, MI-PI-12K, Merck KGaA, Darmstad, Germany) and IGF-1 (mean intra- and inter-assay coefficients of variation were 8.8% and 11.5%, respectively for 60.5 ng/mL; IGF-I RIA-CT, Ref IGF-R22, DiaSorin, Saluggia, Italy) were determined using radioimmunoassays. Blood vitamin B2 (flavin adenine dinucleotide (FAD) and riboflavin), and plasma vitamin B6 (pyridoxal-5’-phosphate (P5P) and pyridoxal), were quantified using the ClinRep Complete kit according to manufacturer’s recommendations (Recipe, Munich, Germany) and extracts were analyzed by UPLC (Waters, Milford, MA, USA) as previously detailed by Meale et al.^6^. Intra- and inter-assay coefficients of variation determined using a reference bovine blood sample analysed repeatedly for 3 years were 3.3 and 17.5% for FAD, 3.3 and 13.4% for riboflavin, 1.0 and 6.1% for P5P, and 2.7 and 6.4% for pyridoxal.

A useful and easy-to-determine index of insulin sensitivity (RQUICKI index) proposed by Holtenius and Holtenius^42^ was estimated. In brief, it was calculated based on plasma concentrations of glucose, insulin and NEFA according to Eq. (1). Increased values of RQUICKI are indicative of increased insulin sensitivity.

1 $$\begin{aligned} RQUICKI=\frac{1}{log[glucose\,(\rm{mg}/\rm{dL})] + log[NEFA\,(\rm{mmol}/\rm{L})] + log [insulin\,(\upmu \rm{U}/\rm{mL})]} \end{aligned}$$

Additionnaly, several ratios between vitamins concentrations reflecting the vitamin activation levels were also determined for vitamins B2 and B6 to state their role in feed efficiency as already explored for vitamin D vitamers and diabetes^60^.

### Targeted metabolomic analysis and data acquisition

Frozen plasma samples were thawed overnight, centrifuged and the supernatant subjected to metabolomic profiling analysis at the core lab of Biocrates Life Science AG (Innsbruck, Austria). A targeted metabolomic approach was carried out using a commercially available kit (MxP Quant 500 kit, Biocrates Life Sciences, Innsbruck, Austria) for the quantification up to 630 metabolites belonging to 26 metabolites classes after derivatization by flow injection analysis-tandem mass spectrometry (FIA-MS/MS) in the case of lipids and hexoses, while small molecules were quantified by liquid chromatography-tandem mass spectrometry (LC-MS/MS). Both FIA-MS/MS and LC-MS/MS were performed using a 5500 QTRAP instrument (AB Sciex, Darmstadt, Germany) with an electrospray ionization (ESI) source. Data were quantified using a specific mass spectrometry software (Sciex Analyst) and further analysed using the Biocrates MetIDQTM. A complete list of analysed metabolites is available in Suppl. Table S1.

A cleaning data procedure was performed on raw data in order to exclude metabolites with missing values or concentration below the limit of detection (LOD). Metabolites were excluded when values were under the LOD in $\ge$ 20% of measured samples within each experimental group (RFI $\times$ Diet combination). In case of missing values among the retained metabolites, imputed values were added by replacing values below LOD by values between LOD and LOD/2 using a logspline imputation method^61^. Finally, the imputed database ($\upmu$mol/L concentration data) was transformed by log 2 in order to avoid heteroscedasticity and any skewed distributions of data^62^.

### Calculations and statistical analysis

Intakes of organic matter, crude protein, neutral detergent fibre and starch were calculated on the basis of the observed DMI and analysed chemical composition of diets as reported in Cantalapiedra-Hijar et al.^26^. Diets were characterized by the INRA feeding system^57^ from analysed chemical composition and observed animal feeding level. Net energy intake and metabolizable protein intake were estimated, as well as rumen fermentation parameters (rumen fermentable OM and NDF) and starch digestibility (starch digested in the small intestine) as they are needed to estimate the net portal appearance of total volatile fatty acids, acetate, propionate, butyrate and glucose according to equations proposed by Loncke et al.^63^.

Prior to statistical analysis, all data -including cleaned metabolomic data set- were adjusted for the effect of the cohort by using a linear model with cohort as the only fixed effect. Adjustment consisted of adding up the average metabolite concentration from the whole population (i.e. intercept) to the individual residuals obtained in that model. Because the obtained residuals represent the variation of each observation from the average of its respective cohort, the between-cohort variation was in this way removed from raw values. Then, nutrient intakes, performance data and classical metabolic parameters were analysed by ANOVA considering diet, RFI group and their interaction as fixed effects. For performance, classical metabolites, hormones and vitamins, the analyses were performed using the R software (R development Core Team, 2015) and significance was set at *P* $\le$ 0.05, and tendency was declared at 0.05 < *FDR* $\le$ 0.10. For metabolomic data, the same univariate analyses were performed using the MetaboAnalyst software^64^ (https://www.metaboanalyst.ca/). The obtained raw-*P* values were adjusted by the Benjamini-Hochberg^65^ false discovery rate (FDR) correction as previously suggested^66^. Significance threshold was set at *FDR* $\le$ 0.05, and tendency was declared at 0.05 < *FDR* $\le$ 0.10. We aimed to compare how Low- and High-RFI metabolite profiles contrasted over the two diets. Therefore, when a significant RFI $\times$ Diet interaction was detected, pairwise comparisons performed by Tukey’s honestly significance difference were only applied within each diet (sliced comparisons) as previously reported^64,67^. Given the relatively small change in the plasma metabolite concentration expected across individuals fed the same diet but differing in RFI, no restrictive threshold fold-change cut-off was applied when interpreting FDR significant metabolites^10,68^.

Metabolomic data was additionally submitted to multivariate analyses. These included both clustering analysis by principal component analysis and classification models assessment by partial-least square discriminant analysis. The quality of prediction from PLS-DA models were evaluated through the *Q*$^2$ criteria. In addition, after univariate analysis, PCA analyses were performed *a posteriori* in order to explore the associations between RFI, and those classical and metabolomic variables with the greater significant differences across RFI groups within each diet. Finally, in order to gain deeper insights on mechanisms underlying RFI differences, metabolic pathways analysis, based on the *Bos taurus* KEGG database (https://www.genome.jp/), were performed combining the *Global test* and a topology analysis based on betweenness centrality^64^. In brief, *Global test* determines whether a set of metabolites that participate in a specific metabolic pathway present or not a global trend when comparing two treatments. Finally, it gives a global *P* value for the given metabolic pathway after the evaluation of the effect of treatment on each metabolite^69^. In addition, betweenness centrality estimates how frequently a node (a given compound) is on the shortest pathways between every pair of compounds for detecting bottlenecks in a network. In other words, it gives an idea of how well interconnected (short biochemical connections) are the compounds measured for each given metabolic pathway^64^. Significant enrichment of metabolic pathway was set at *FDR* $\le$ 0.05, and only metabolic pathways with at least two metabolites quantified in the current data set were further considered for discussion purposes.

## Supplementary information

Supplementary Information 1.

Supplementary Information 2.
