# Supplementary material for: Common and diet-specific metabolic pathways underlying residual feed intake in fattening Charolais yearling bulls
Source: Sci Rep. 2021 Dec 21;11:24346. doi: 10.1038/s41598-021-03678-x (PMC8692463; doi:10.1038/s41598-021-03678-x)
Supplement: Supplementary file 2 — Supplementary Information 2. [file 41598_2021_3678_MOESM2_ESM.pdf]

Common and diet-specific metabolic pathways underlying residual feed intake in fattening Charolais yearling bulls

Ezequiel Jorge-Smeding[1], Muriel Bonnet[2], Gilles Renand[3], Sébastien Taussat[3,4], Benoit Graulet[2], Isabelle Ortigues-Marty[2], and Gonzalo Cantalapiedra-Hijar[2]

1: Universidad de la República, Facultad de Agronomía, Departamento de Producci ón Animal y Pasturas, Av. Garzón780, Montevideo, Uruguay
2: INRAE, Université Clermont Auvergne, UMRH, 63122, Saint-Genes-Champanelle, France
3: INRAE, AgroParisTech, Université Paris-Saclay, GABI, 78350, Jouy-en-Josas, France
4: Allice, 149 rue de Bercy, 75595 Paris Cedex 12
*Corresponding author: gonzalo.cantalapiedra@inrae.fr

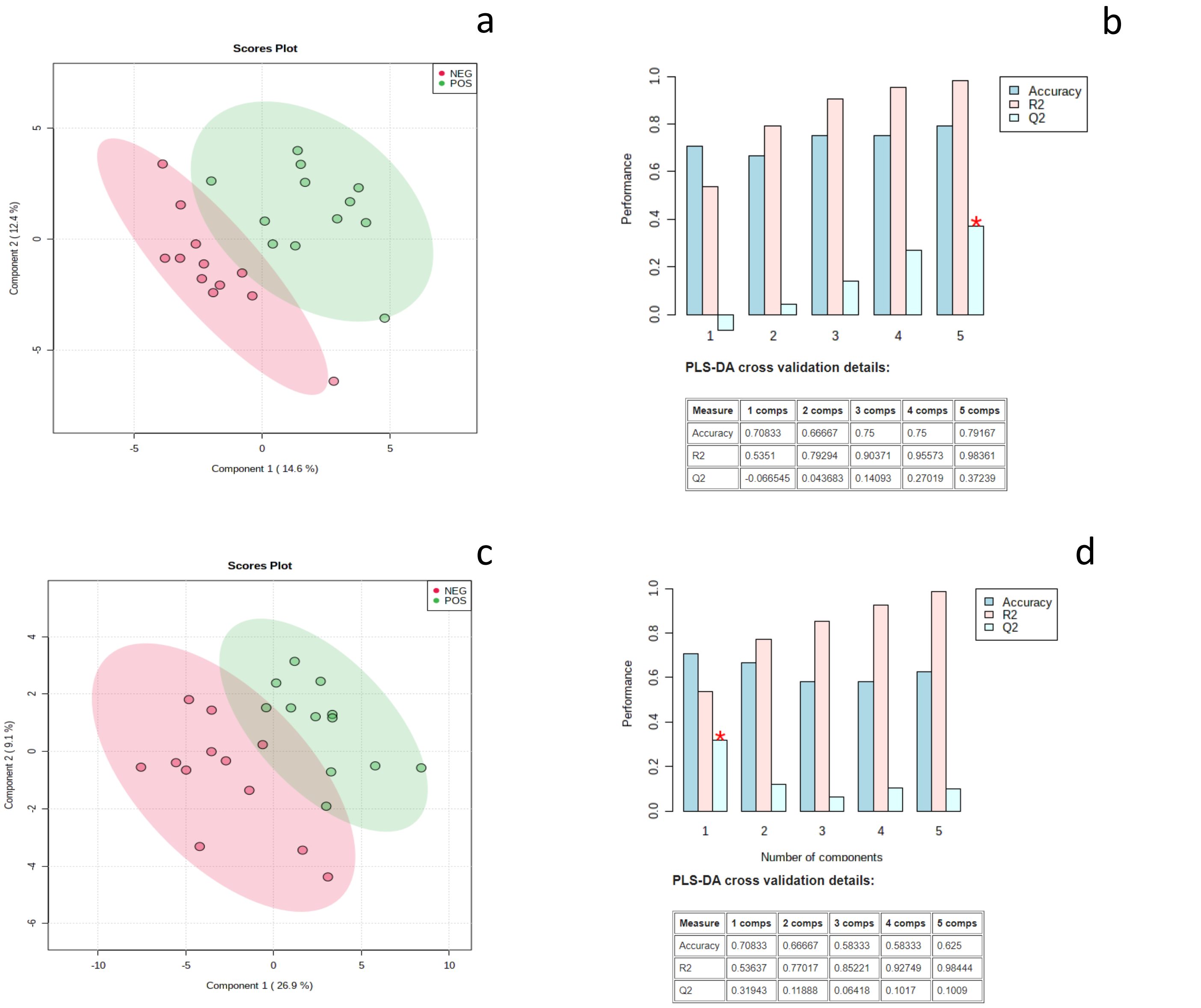

Measure	1 comps	2 comps	3 comps	4 comps	5 comps
Accuracy	0.70833	0.66667	0.75	0.75	0.79167
R2	0.5351	0.79294	0.90371	0.95573	0.98361
Q2	-0.066545	0.043683	0.14093	0.27019	0.37239

Measure	1 comps	2 comps	3 comps	4 comps	5 comps
Accuracy	0.70833	0.66667	0.58333	0.58333	0.625
R2	0.53637	0.77017	0.85221	0.92749	0.98444
Q2	0.31943	0.11888	0.06418	0.1017	0.1009

Supplemtary Fig. S1. Partial least square discriminant analysis (PLS-DA) models and cross validation criteria for corn- (a, b) and grass-silage (c, d) diets based on all quantified metabolites.
